# Assessment of different preservation techniques for human skeletal muscle biopsy samples: A comparative method study on freeze‐drying, RNAlater, and RNAlater‐ICE

**DOI:** 10.14814/phy2.70562

**Published:** 2025-09-10

**Authors:** Sebastian Edman, Alice Engvall, Tuva Eriksson Viklund, Oscar Horwath, William Apró

**Affiliations:** ^1^ Department of Physiology, Nutrition and Biomechanics Swedish School of Sport and Health Sciences Stockholm Sweden; ^2^ Department of Women's and Children's Health Karolinska Institutet Stockholm Sweden; ^3^ Department of Clinical Science, Intervention and Technology Karolinska Institutet Stockholm Sweden

**Keywords:** enzyme activity, freeze‐drying, glycogen, lyophilization, muscle biopsy, post‐translational modifications, RNAlater, RNAlater‐ICE, Western blot

## Abstract

Human skeletal muscle comprises slow‐twitch (type I) and fast‐twitch (type II) fibers. Fiber type‐specific analyses often require manual isolation of fibers, necessitating effective tissue preservation. While freeze‐drying remains the standard, alternative preservation methods such as RNAlater and RNAlater‐ICE are increasingly used. Besides their utility in preserving RNA, it needs to be determined whether RNAlater and RNAlater‐ICE can be utilized for broader downstream biochemical analyses in skeletal muscle tissue. In this study, we compared freeze‐drying to RNAlater and three RNAlater‐ICE‐based protocols. We observed substantial and consistent alterations in protein content, amino acid levels, and enzyme activity depending on the preservation method. Notably, all RNAlater‐ICE protocols abolished citrate synthase activity, and branched‐chain amino acid levels were markedly reduced in both RNAlater and RNAlater‐ICE‐treated samples relative to freeze‐dried tissue. Total protein concentration was comparable between freeze‐dried and RNAlater‐preserved muscle, whereas RNAlater‐ICE protocols yielded lower values. After centrifugation, supernatant protein concentration was higher in RNAlater‐treated samples, but consistently lowest following RNAlater‐ICE treatment. Our results demonstrate the importance of choosing an appropriate preservation method for skeletal muscle prior to downstream biochemical analysis and that care should be taken when using RNAlater and RNAlater‐ICE for protein or amino acid analysis.

## 1 INTRODUCTION

Skeletal muscle is a dynamic and highly malleable tissue composed of different cell types, that is, fiber types. In adult human muscle, three main fiber types have been categorized based on myosin heavy chain (MyHC) isoform expression: type I, type IIA, and type IIX (Schiaffino & Reggiani, 2011). Each of these fiber types has unique functional and metabolic properties (Essen et al., 1975). For instance, type I fibers contract at slow speeds, are resistant to fatigue, and have a large capacity for insulin‐mediated glucose uptake. In contrast, type II fibers have high peak contraction speed, fast glycogen turnover, and rely heavily on anaerobic metabolism (Albers et al., 2015; Bottinelli et al., 1991; Edström & Kugelberg, 1968). Fiber type composition is not only a key determinant of muscle function, but fiber type‐specific properties have in several reports been linked to metabolic variation also at the whole organism level, such as insulin‐mediated glucose uptake, blood pressure, and indices of obesity (Blackwood et al., 2022; Damer et al., 2022; Hernelahti et al., 2005; Lillioja et al., 1987; Marin et al., 1994; Tanner et al., 2002). Thus, insights into the physiology of different fiber types provide information relevant to both health and disease.

Analyses of skeletal muscle on the fiber type‐specific level can be performed using different methods, one of which is by isolating individual muscle fibers (Tobias & Galpin, 2020). To enable muscle fiber isolation, freeze‐drying is commonly used as a preservation method. This cost‐effective technique has been applied to muscle biopsy material for over 50 years and remains compatible with most of the analyses performed today (Albers et al., 2015; Deshmukh et al., 2021; Edman et al., 2019; Jonsson et al., 2022; Wyckelsma et al., 2020). By removing solvents (water) and oxygen from the sample under low pressure, freeze‐drying prevents the decay of biological tissues without affecting sensitive cellular processes, such as enzyme activity and post‐translational modifications (Damsteegt et al., 2016; Molnar et al., 2021; Wu et al., 2012). This represents a practical advantage during fiber isolation and processing since freeze‐dried muscle samples can be handled at room temperature without cooling systems or buffering solutions.

A commonly reported caveat of freeze‐drying is that the treatment embrittles the structure of the tissue. This makes the muscle fibers more prone to breaking during isolation and limits the ability to isolate intact fibers of sufficient size, which sometimes is a necessity to provide enough material for both fiber type identification and downstream analyses, as discussed previously (Horwath, Edman, et al., 2022). Although a skilled technician can mitigate this issue, it further strains an already time‐consuming analysis and limits a high‐throughput workflow. Hence, alternative preservation strategies need to be assessed to make fiber type‐specific investigations more time‐efficient.

RNAlater is a preservation reagent designed to stabilize and protect RNA from degradation (Bennike et al., 2016; Kasahara et al., 2006; Mutter et al., 2004; van Eijsden et al., 2013). This medium was initially intended to preserve unfrozen tissue specimens, but it can also be applied to frozen tissue in the form of RNAlater‐ICE (Mutter et al., 2004). Independent of which form the RNAlater comes in, the underlying principle is to prevent RNA degradation by rapidly permeating the tissue with high concentrations of ammonium and cesium sulfates (RNAlater), which are agents known to denature DNases, RNases, and proteases (Allewell & Sama, 1974), or a mixture of alcohols (RNAlater‐ICE). Unlike freeze‐drying, tissue preserved in RNAlater solution remains flexible and resilient to manual isolation after the treatment. This represents a significant advantage as individual muscle fibers can be isolated without risking their structural integrity (Galpin et al., 2012; Tobias et al., 2020). In turn, this allows for a faster process and reduces the number of fibers that need to be discarded throughout the dissection process. However, before RNAlater variants can be routinely used for this purpose, it is critical to evaluate the compatibility of these compounds with biochemical methods commonly used in muscle physiology research. While some studies reported that RNAlater is effective for preserving RNA integrity in various tissues (Kasahara et al., 2006; van Eijsden et al., 2013), others have raised concerns as the treatment leads to alterations in protein extraction and preservation of post‐translational modifications (Kruse et al., 2017). Some studies have used different RNAlater variants together with various lysis buffers for analyzing muscle samples (Galpin et al., 2012; Raue et al., 2024; Tobias et al., 2018); however, to date, no systematic comparisons have been made to evaluate their utility in preserving skeletal muscle tissue.

Thus, the present study aimed to determine whether RNAlater and RNAlater‐ICE are valid alternatives to freeze‐drying for preserving muscle biopsies. More specifically, we aimed to compare freeze‐dried biopsy samples to samples preserved either in RNAlater or in RNAlater‐ICE on measures of global protein concentration, glycogen content, amino acid concentrations, citrate synthase activity, and protein extraction.

## 2 MATERIALS AND METHODS

### 2.1 Ethical approval

Muscle biopsy samples used for experiments in the present study were obtained from an ongoing project with ethical approval from the Swedish Ethical Review Authority (DNR 2019‐02194). The study was conducted in line with the principles outlined in the Declaration of Helsinki, and all participants gave oral and written consent after being informed about the possible risks associated with each study.

### 2.2 Muscle biopsy sampling and tissue preservation

**FIGURE 1:**
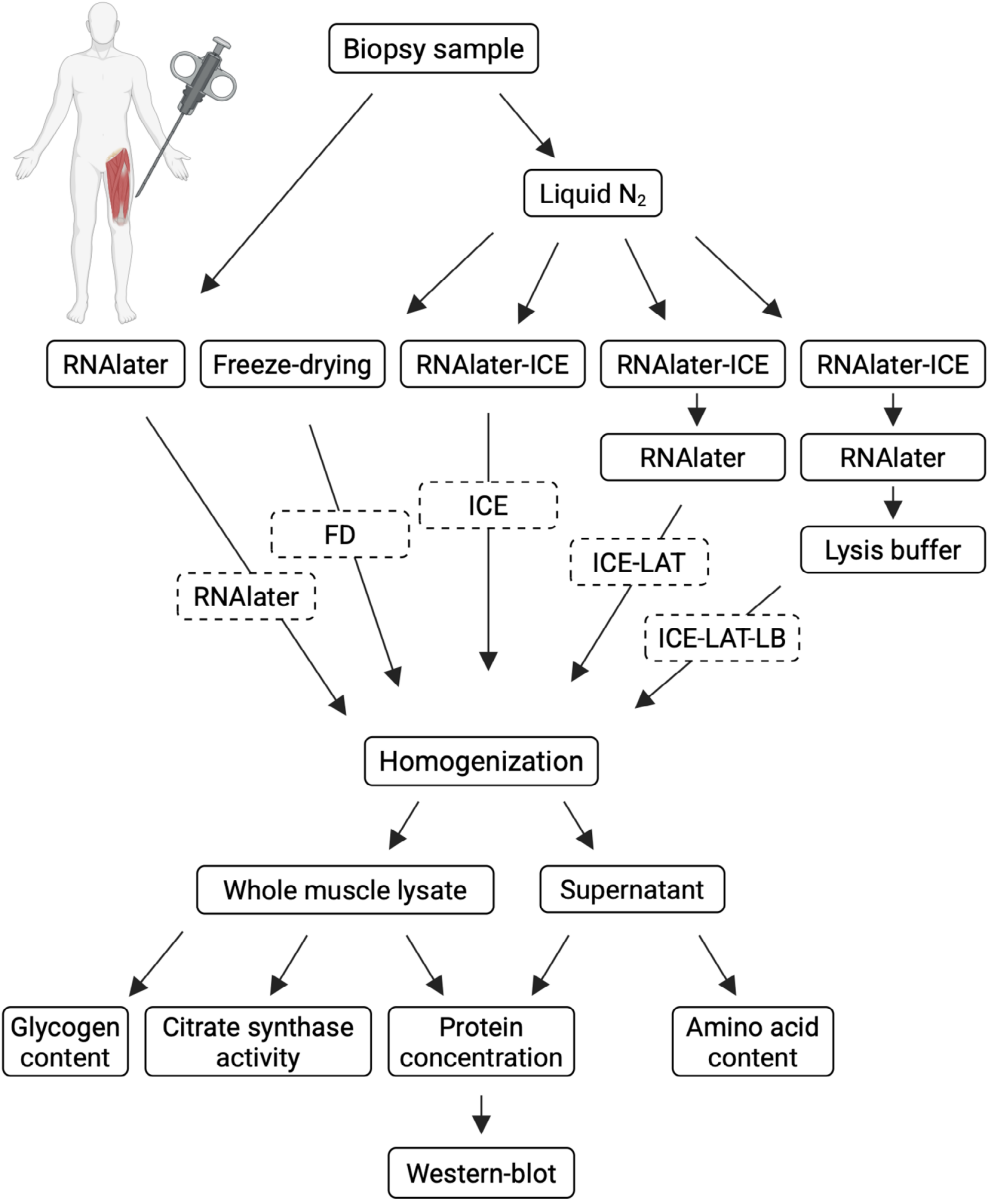
Schematic outline of the muscle biopsy handling. Full lines indicate the treatment and techniques used. Dashed lines indicate sample naming.

Muscle samples were obtained in the rested and fasted state from three physically active and healthy individuals (age 25–31 years). After the administration of local anesthesia (Carbocain 20 mg mL^−1^, AstraZeneca, AB, Södertälje Sweden), a biopsy was collected from the *vastus lateralis* muscle using a Bergström needle with manual suction applied. Immediately after excision, the wet weight of the muscle samples was determined using a laboratory microbalance (Cubis MCA2.7S‐2S00‐M, Sartorius Lab Instruments GmbH & Co, Göttingen, Germany). The samples were then rapidly minced and mixed with a scalpel and divided into five separate aliquots. These were reweighed and transferred to new tubes for subsequent treatment. One aliquot was immediately submerged in 10 volumes of precooled RNAlater solution (Thermo Scientific, Rockford, USA; Cat# AM7020) and kept at +4°C for 48 h and then stored at −30°C. The remaining aliquots were snap‐frozen in liquid nitrogen and stored at −80°C until treatment. One of these aliquots was freeze‐dried overnight, while the remaining three aliquots were placed in 10 volumes of precooled RNAlater‐ICE solution (Thermo Scientific; Cat# AM7030) and kept at −30°C for 48 h. Two of these aliquots were then transferred to new tubes containing 100 μL of precooled RNAlater solution and kept at −30°C for 16 h. Lastly, one of these aliquots was transferred to a new tube containing lysis buffer without Triton X‐100 (see below) and kept on ice for 2 h. Muscle samples were carefully blotted on paper tissue between each transfer step to minimize the carryover of one solution into another. All treatments were timed so that all aliquots from one tissue sample could be homogenized at the same time. See Figure 1 for more details.

### 2.3 Homogenization and sample preparation

Procedures related to homogenization, sample preparation, and immunoblotting have been described previously (Apro et al., 2015; Horwath, Moberg, et al., 2022). Briefly, samples were homogenized in ice‐cold lysis buffer (100 μL/mg of estimated dry weight) using a Bullet Blender (Next Advance, USA) and 0.5 mm Zirconium Oxide beads. The lysis buffer had a final concentration of 40 mM HEPES (pH 7.5), 1 mM EDTA, 120 mM NaCl, 10 mM β‐glycerophosphate, 10 mM Na_4_P_2_O_7_, 50 mM NaF, 0.25% Triton X 100, 1% phosphatase inhibitor cocktail (Sigma P0044), and 1% (vol/vol) Halt Protease Inhibitor Cocktail (Thermo Scientific, Cat# 78430). Following 8 × 1 min in the Bullet Blender, samples were shaken (2200 rpm) for 1 h on an orbital shaker. The crude lysate was then aliquoted for analysis of citrate synthase activity, glycogen content, and immunoblotting. In addition, one aliquot was centrifuged at 10,000 *g* for 10 min to generate a supernatant for amino acid analyses and immunoblotting. Protein concentration was determined in both the lysate and the supernatant using Pierce 660 nm protein assay (Thermo Scientific, Cat# 22662). For immunoblotting, lysis buffer and 4× Laemmli sample buffer (Bio‐Rad Laboratories, Richmond, USA, Cat# 1610747) were combined to adjust the protein concentration to 1.0 μg μL^−1^ and 0.5 μg μL^−1^ for whole muscle lysates and supernatant, respectively. The samples were then heated for 30 min at 37°C and subsequently stored at −80°C until analysis.

### 2.4 Citrate synthase activity

Citrate synthase activity was determined as described previously (Alp et al., 1976). In brief, 5 μL of crude lysate was combined with 900 μL of assay buffer, after which the reaction was initiated by the addition of 100 μL of oxaloacetate. As a blank, 5 μL of homogenization buffer was used. Final concentrations in the assay buffer were 50 mM Tris–HCL (pH 7.5), 0.1 mM Acetyl‐CoA, 0.2 mM (DTNB; (5,5‐dithiobis (2‐nitrobenzoic acid))), and 0.5 mM oxaloacetate. Absorbance following the conversion of DTNB to thionitrobenzoate as the end product of the assay was measured at 412 nm on a spectrophotometer (DU800, Beckman Coulter AB, Bromma, Sweden). Citrate synthase activity was measured under linear conditions (8 min).

### 2.5 Glycogen content

Extraction of muscle glycogen and subsequent digestion into free glucose was performed as previously described (Harris et al., 1974), with minor modifications. Briefly, 20 μL crude lysate was combined with equal amounts of 2 M KOH and heated for 30 min at 80°C with occasional vortexing. Following cooling to room temperature, samples were diluted 10‐fold by the addition of 360 μL of 1 M KOH. An aliquot of 20 μL of each diluted sample was then combined with 40 μL neutralization solution (equal amounts of 15 mM sodium acetate buffer, pH 4.9 and 0.5 M HCl). Next, 0.42 units of amylglucosidase in 10 μL of acetate buffer (15 mM sodium acetate, pH 4.9) was added to all neutralized samples, which were then incubated overnight at 40°C on an orbital shaker.

The amount of liberated glucose following amyloglucosidase digestion was measured fluorometrically using an enzymatic assay with hexokinase and glucose‐6‐phosphate dehydrogenase (G6PDH) as outlined previously (Lowry & Passonneau, 1972), again with some minor modifications. First, 10 μL of the digested sample was loaded in triplicate on a black 96‐well plate. Next, 195 μL assay buffer ([Final] 100 mM triethanolamine (pH 7.8), 4 mM MgCl_2_, 0.5 mM EDTA, 0.5 mM dithiothreitol, 0.2 mM NADP, and 2 mM ATP) and 0.5 U/mL G6PDH was added to each well. After mixing, a blank reading was obtained for each sample, after which the assay reaction was initiated by the addition of 5 μL of hexokinase ([final] 0.57 U/mL) to each well. The conversion of NADP to NADPH was monitored fluorometrically (excitation 360 nM/emission 460 nM) on a plate reader (Infinite F200 Pro, Tecan, Männedorf, Switzerland) for 15 min. Glucose concentrations were calculated based on a standard curve for NADH.

### 2.6 Amino acid concentrations

Before measuring amino acid concentrations, samples were deproteinized by combining 20 μL of supernatant with equal volumes of trichloroacetic acid (5% w/v) and incubated for 30 min on ice. The samples were then centrifuged at 10,000 *g* at 4°C for 10 min. The deproteinized supernatant containing free amino acids was then transferred to new tubes and stored at −80°C until analysis. Before analyses, samples, together with a standard curve, were derivatized using the AccQ Tag Ultra Derivatization Kit (Waters Sverige AB, Solna, Sweden, Cat# 186003836) according to the manufacturer's instructions. Amino acid concentrations were then measured using high‐specificity quantitative liquid chromatography–tandem mass spectrometry (Xevo™ TQ MS, Waters).

### 2.7 SDS‐PAGE and immunoblotting

From each sample, 10 μg and 5 μg of protein from the lysate and the supernatant were loaded on 18‐well Criterion TGX gradient gels (4%–20% acrylamide; Bio‐Rad Laboratories, Cat# 5678094), respectively. Electrophoresis was performed on ice for ~30 min at 300 V. The gels were incubated for 30 min in transfer buffer (25 mM Tris base, 192 mM glycine, and 10% methanol) before the proteins were transferred onto PVDF membranes (Bio‐Rad Laboratories, Cat# 1620177) at constant current (300 mA) for 180 min on a bed of ice. MemCode™ Reversible Protein Stain Kit (Thermo Scientific, Cat# 24580) was used to confirm even transfer of proteins on the membranes. After destaining, membranes were blocked for 1 h in Tris‐buffered saline (TBS; 20 mM Tris base, 137 mM NaCl, pH 7.6) containing 5% nonfat dry milk followed by overnight incubation with primary antibodies diluted in TBS supplemented with 0.1% Tween‐20 and 2.5% nonfat dry milk (TBST‐M). The next morning, membranes were washed and incubated with species‐specific secondary antibodies conjugated to horseradish peroxidase for 1 h. Membranes were thereafter washed again (TBST) followed by incubation with Super Signal™ Femto Chemiluminescent Substrate (Thermo Scientific, Cat# 34095) to visualize bands in the molecular imager (ChemiDoc™MP, Bio‐Rad Laboratories).

### 2.8 Antibodies

For immunoblotting, primary antibodies against mTOR (#2983, 1:1000), S6K1 (#2708, 1:1000), eEF2 (#2332, 1:1000) CS (#96600, 1:1000), Pan Actin (#8456S, 1:10,000), and p‐eEF2 (#2331, 1:1000) were purchased from Cell Signaling Technology (Beverly, USA). The primary antibody against RPS6 (#sc‐74459, 1:500) was purchased from Santa Cruz Biotechnology (Heidelberg, Germany). Anti‐fast Myosin (ab91506, 1:20000) and NDUFB8 (ab110242, 1:10,000) were purchased from Abcam (Cambridge, UK). Secondary anti‐mouse (#7076, 1:10,000) and secondary anti‐rabbit (#7074, 1:10,000) were purchased from Cell Signaling Technology.

### 2.9 Statistics

A Friedman's repeated measures ANOVA with Durbin‐Conover pairwise comparisons post hoc was used. The level of significance was set at *p* < 0.05. Statistical analysis was conducted using Jamovi (Version 2.5).

## 3 RESULTS

**FIGURE 2:**
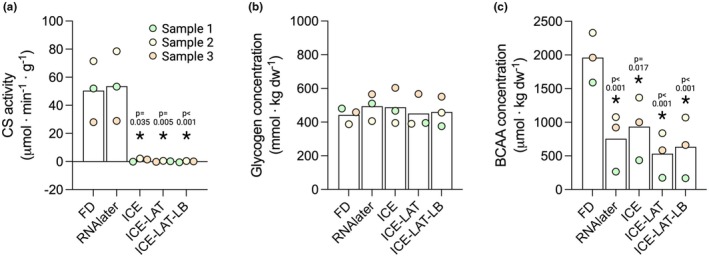
The effect of different muscle biopsy preservation techniques on enzyme activity and metabolite extraction. (a) Citrate synthase activity, (b) Muscle glycogen concentration, **c**) Branched‐chain amino acid concentration, BCAA, branched‐chain amino acids; FD, freeze‐dried, ICE, RNAlater‐ICE; ICE‐LAT, RNAlater‐ICE plus RNAlater; ICE‐LAT‐LB, RNAlaterICE plus RNAlater and Lysis buffer. Statistical analysis by Friedmans ANOVA with Durbin–Conover pairwise comparisons post hoc, *p < 0.05 versus FD, colors indicate separate samples. *N* = 3.

**FIGURE 3:**
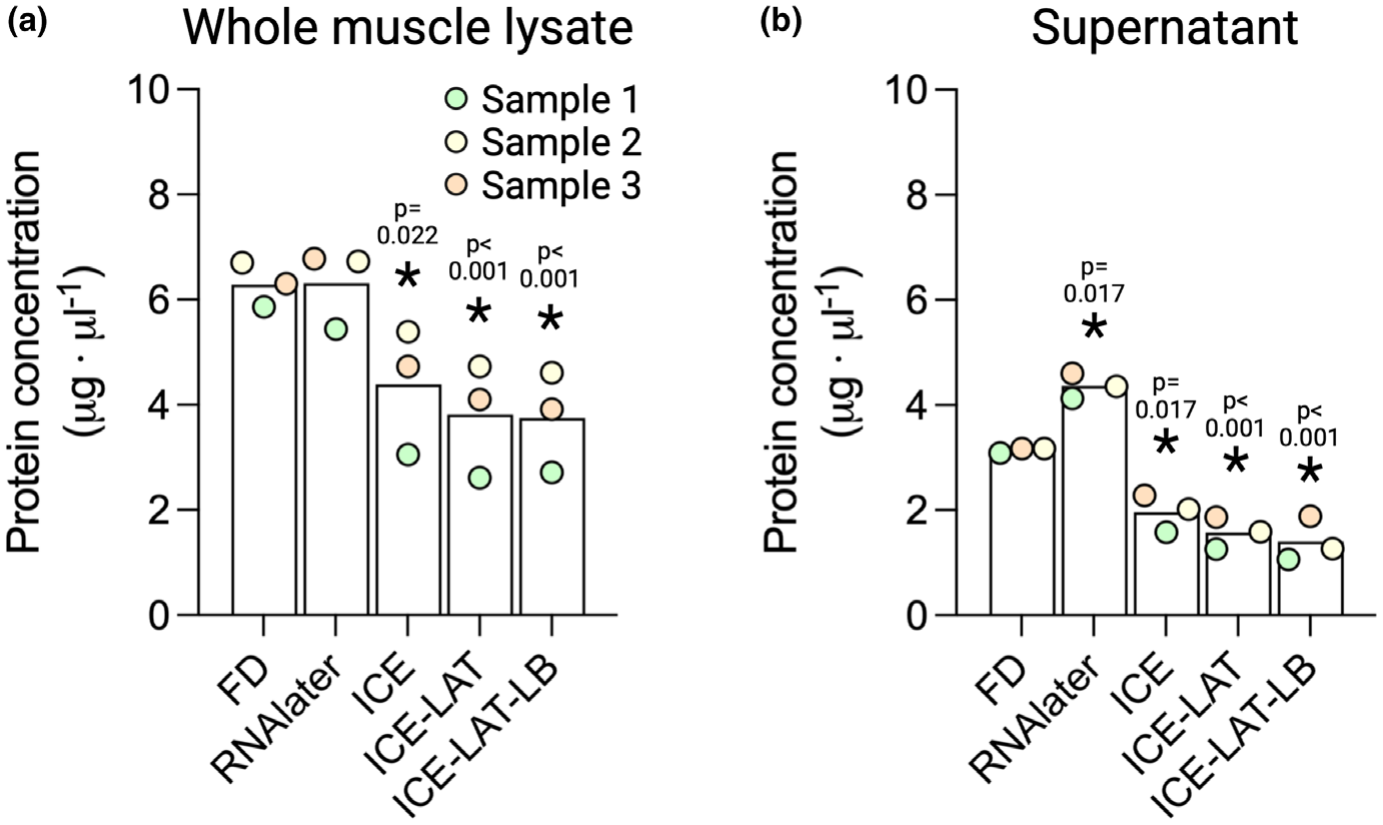
The effect of different muscle biopsy preservation techniques on protein concentration. (a) Protein concentration in crude lysate, (b) Protein concentration in supernatant. FD, freeze‐dried; ICE, RNAlater‐ICE; ICE‐LAT, RNAlater‐ICE plus RNAlater; ICE‐LAT‐LB, RNAlaterICE plus RNAlater plus Lysis buffer. Statistical analysis by Friedmans ANOVA with Durbin‐Conover pairwise comparisons post hoc, *p < 0.05 versus FD, colors indicate separate samples. *N* = 3.

In this study, we evaluated how preservation of muscle tissue in two different commercial solutions would affect several biochemical analyses commonly performed in skeletal muscle, in comparison to classical freeze‐drying. Citrate synthase activity was similar between samples preserved by freeze‐drying and RNAlater (51 and 54 μmol · min^−1^ · g^−1^, respectively, *p* = 0.094); however, all variants of the RNAlater‐ICE treatment completely abolished citrate synthase activity (0.0–1.1 μmol · min^−1^ · g^−1^, *p* = 0.035‐*p* < 0.001 vs. FD; Figure 2a). In contrast, glycogen concentration was similar (442–494 mmol · kg dw^−1^) in all samples regardless of the preservation method used (Figure 2b). Compared to FD, intracellular concentrations of the branched‐chain amino acids were significantly reduced (52%–73% vs. FD, *p* = 0.017‐*p* < 0.001) by all RNAlater (p < 0.001)/RNAlater‐ICE treatments (*p* = 0.017‐*p* < 0.001; Figure 2c). Protein concentrations in the crude lysate were similar in freeze‐dried and RNAlater‐preserved samples (6.29 and 6.32 μg · μL^−1^, respectively) while all variants of the RNAlater‐ICE treatment yielded significantly lower protein concentrations (30%–41%; *p* = 0.022‐*p* < 0.001, Figure 3a) compared to FD. In the supernatant, protein concentration was significantly higher in the RNAlater treatment (+39% vs. FD, *p* = 0.017) while all RNAlater‐ICE treatments had a similar effect on protein concentrations as in the crude lysate, with 38%–56% reductions observed compared to FD (*p* = 0.017‐*p* < 0.001, Figure 3b). Western blot outcomes were only conducted as supporting and exploratory analyses and therefore not quantified.

## 4 DISCUSSION

Here we compared the utility of two commercially available solutions for preserving biochemical properties in human skeletal muscle samples. RNAlater and RNAlater‐ICE are reliable methods for preserving RNA integrity in biological samples (Mutter et al., 2004). Following treatment with RNAlater variants, skeletal muscle retains its structural integrity, simplifying single fiber isolation for downstream fiber type‐specific analysis (Galpin et al., 2012). However, systematic investigations of RNAlater variants for tissue preservation are scarce, and the results are so far inconclusive concerning their utility outside the preservation of RNA (Bennike et al., 2016; Kruse et al., 2017; Mutter et al., 2004; Zhu et al., 2019). More specifically, no previous studies have investigated how RNAlater variants affect enzyme function and metabolite extraction from muscle tissue.

**FIGURE 4:**
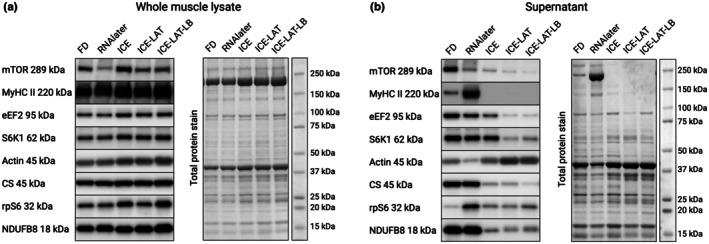
The effect of normalizing protein content based on proteins concentrations after use of different preservation techniques. (a) Content of various individual proteins (left) and total protein (right) in the crude lysate and (b) content of various individual proteins (left) and total protein (right) in the supernatant. FD, freeze‐dried; ICE, RNAlater‐ICE; ICE‐LAT, RNAlater‐ICE plus RNAlater; ICE‐LAT‐LB, RNAlaterICE plus RNAlater plus Lysis buffer. Blots represent one sample.

Freeze drying has been shown to preserve the activity of various metabolic enzymes (Meijer et al., 1977; Wu et al., 2012), including that of citrate synthase (Flockhart et al., 2021; Horwath, Edman, et al., 2022). Here, we found that the enzymatic activity of citrate synthase was unaffected by RNAlater preservation (Figure 2a). In contrast, preservation with all three variants using RNAlaterICE completely abolished citrate synthase activity (Figure 2a). Interestingly, this appears to be a direct result of the compounds found in RNAlaterICE, as the protein content of citrate synthase in the crude lysate was fully maintained in all three variants (Figure 4a). It is important to note that despite similar commercial names, RNAlater and RNAlaterICE are composed of different compounds, with the latter containing a mixture of ethyl‐, methyl‐, and propyl‐alcohol. It is well established that these alcohols have a denaturing effect on various proteins (Bull & Breese, 1978), which likely explains the complete lack of citrate synthase activity in the RNAlaterICE aliquots. Importantly, subsequent serial incubation in RNAlater (ICE‐LAT) and lysis buffer (ICE‐LAT‐LB) did not counteract the denaturing effect of RNAlaterICE.

In contrast, none of the RNAlater variants had a negative impact on the analysis of glycogen content (Figure 2b). Based on the results of the citrate synthase assay, one might have expected that at least the RNAlaterICE variants would have shown lower glycogen content. Mechanistically, this would have occurred if the various alcohols present in RNAlaterICE had denatured the enzymes employed in the glycogen assay. As such, inhibition of the assay enzymes would have yielded a lower amount of free glucose by amyloglucosidase, as well as lower NADH by glucose‐6‐phosphate dehydrogenase to be detected in the assay. However, in the first step, when amyloglucosidase is added to the neutralized lysate, there is a 70‐fold dilution of the original crude lysate obtained after homogenization. This dilution is further increased approximately 20‐fold in the second step, when hexokinase and glucose‐6‐phosphate dehydrogenase are added to the assay buffer. Consequently, any residual alcohol remnants were most likely too low to exert any inhibitory effects in the assay.

Small‐sized, single‐unit metabolites, such as free amino acids, are commonly extracted from freeze‐dried muscle with single‐step protocols where the tissue is submerged in various acid or alcohol solutions without the need to completely dissolve the tissue sample (Vesali et al., 2002). The membrane‐permeating properties of alcohols are well‐known and exploited in various products and research protocols. These include disinfectants, which kill bacteria through membrane disruption (Goldstein, 1986), and imaging protocols where alcohols are used to permeabilize cell membranes to allow antibody entry to the cell interior (Yuan et al., 2017). Based on these properties, one would expect amino acids to leak into the RNAlaterICE solutions, but whether this would also occur with salt‐based RNAlater was less clear. Here we found that amino acid concentrations were similarly reduced with all four RNAlater variants compared to freeze‐drying (Figure 2c). These findings indicate that salt‐based RNAlater has similar membrane‐permeating properties as the mixture of alcohols present in RNAlaterICE. Interestingly, though, multiple transfers between solutions (ICE‐LAT and ICE‐LAT‐LB) did not further decrease amino acid content in the muscle samples. This indicates that the major portion of amino acids leaks into the solution in the first submersion. It is also interesting to note that none of the RNAlater variants resulted in complete amino acid depletion, despite multiple transfers between solutions. The reason for this is not fully clear but may be related to passive diffusion of amino acids due to lack of physical pressure during tissue submersion. While traditional amino acid extraction from freeze‐dried muscle does not necessitate homogenization, manual pressure is often applied to squeeze out the amino acids. Regardless, our results show a significant leakage of amino acids into the preservation media when using RNAlater and RNAlaterICE variants.

Determining protein concentration is commonly performed for a variety of biochemical assays but is especially critical when equal amounts of protein are to be analyzed, for instance in SDS‐PAGE and Western blot. Here, we found that the protein concentration of the crude lysate was highly similar when the muscle was preserved by freeze‐drying and in RNAlater. In contrast, the protein assay showed significantly lower concentrations in all three RNAlaterICE variants (Figure 3b). At first glance, these findings indicate that the alcohol mixture found in RNAlaterICE reduces protein extraction following homogenization. However, the same lysis buffer was used for all samples, and in equal volumes per sample weight. Furthermore, the crude lysate was not centrifuged, which in turn prevented any physical loss of sample. Consequently, one would expect similar protein content in all aliquots, yet the protein assay showed 30%–41% lower protein concentration for the RNAlaterICE variants. This rather suggests that the alcohol content of RNAlaterICE may have artefactually influenced the protein assay, potentially through the well‐known protein precipitating effect of various alcohols (Yoshikawa et al., 2012). Indeed, while visual examination of the different lysates showed no signs of intact tissue following homogenization, all RNAlaterICE aliquots had clearly visible precipitates. The colorimetric assay used here contains a dye that reacts with basic residues within proteins. Upon binding to the residues, the dye shifts color in proportion to the amount of protein present in the sample. However, when multiple protein molecules aggregate during precipitation, more of the dye‐binding residues become hidden within the precipitates. The dye will therefore bind to fewer exposed residues, leading to lower absorbance values. When compared to standards without precipitations present, this will be interpreted as lower protein concentration, despite the actual protein content being similar. To determine if protein concentration was indeed underestimated in the protein assay, all samples were subjected to SDS‐PAGE and immunoblotting for a wide range of proteins of different molecular weights. As shown in Figure 4a, the total protein stain clearly shows higher protein content in the lanes of all three RNAlaterICE variants. The larger amount loaded was also reflected in stronger immunostaining signals for most of the proteins analyzed. These findings confirm that preservation in RNAlaterICE can introduce artifacts in downstream analyses such as protein assays.

In many cases, crude lysates are cleared by centrifugation to remove insoluble cellular proteins and structures, often referred to as cellular debris. By pelleting these cellular components, the remaining soluble proteins make up a significantly larger portion of the total protein pool of the resulting supernatant. This will increase the immunostaining signal when equal amounts of total protein are loaded, compared to a crude lysate, and is especially important for very low abundance proteins. While the advantage of clearing the lysate by centrifugation is obvious, it remains unclear how the soluble protein pool is influenced by RNAlater and RNAlaterICE preservation. As expected, the supernatant of all aliquots had lower protein concentration compared to the crude lysate (Figure 3a,b). Interestingly, all RNAlaterICE variants had significantly lower protein concentrations compared to the freeze‐dried aliquot. Importantly, following centrifugation, there were no visible precipitates in the supernatant. As such, lower concentrations are unlikely to be the result of an artifact in the protein assay; rather, it indicates that a larger proportion of the proteins present in the crude lysate of all RNAlaterICE variants were pelleted during centrifugation. Surprisingly, the RNAlater aliquot showed a significantly higher concentration compared to freeze‐drying. This, in turn, suggests that a larger portion of the total protein pool was solubilized when tissue was preserved in RNAlater. To verify this, SDS‐PAGE and subsequent immunoblotting were carried out on the supernatant aliquots as well. From the total protein stain (Figure 4b), it is clear that a large portion of proteins were removed during centrifugation in the RNAlaterICE aliquots, which was again largely reflected in the immunoblots. However, different proteins appear to have been affected in different ways and to a different extent. Interestingly, the most prominent effect was seen for myosin, which was completely removed from the supernatant. Given the extremely high abundance of myosin in skeletal muscle, the complete removal of this protein largely explains the very low protein concentrations in the RNAlaterICE variants. In contrast, preservation in RNAlater resulted in higher myosin content in the supernatant compared to freeze‐drying. This suggests that the higher salt concentration in this aliquot increased the solubility of myosin, but also ribosomal protein S6. Intriguingly, RNAlater also appears to rescue some of the insolubility imposed by previous preservation in RNAlaterICE, as shown by a higher actin content in the ICE‐LAT and ICE‐LAT‐LB aliquots (Figure 4b).

The results presented here show that different preservation methods can have a large and highly variable impact on analytical outcomes and, more importantly, on data interpretation. It is therefore crucial to evaluate the utility of the chosen method for each specific downstream analysis. We suggest that RNAlaterICE may not be a suitable preservation technique when enzymatic activity assessment, or protein and metabolite extraction are to be conducted. Moreover, care should be taken when utilizing RNAlater prior to protein and metabolite extraction from muscle samples.

## FUNDING INFORMATION

This work was supported by project grants (P2020‐0058, P2021‐0173) and an Early Career Research Fellowship (No. D2019‐0050), both from the Swedish National Council for Sport Science awarded to W.A.

## CONFLICT OF INTEREST STATEMENT

The authors have no known competing financial interests or personal relationships that could have appeared to influence the work reported in this paper.

## Data Availability

Data will be available from the authors upon reasonable request.

## References

[phy270562-bib-0001] Albers, P. H. , Pedersen, A. J. , Birk, J. B. , Kristensen, D. E. , Vind, B. F. , Baba, O. , Nohr, J. , Hojlund, K. , & Wojtaszewski, J. F. (2015). Human muscle fiber type‐specific insulin signaling: Impact of obesity and type 2 diabetes. Diabetes, 64, 485–497. 10.2337/db14-0590 25187364

[phy270562-bib-0002] Allewell, N. M. , & Sama, A. (1974). The effect of ammonium sulfate on the activity of ribonuclease a. Biochimica et Biophysica Acta, 341, 484–488. 10.1016/0005-2744(74)90240-x 4838163

[phy270562-bib-0003] Alp, P. R. , Newsholme, E. A. , & Zammit, V. A. (1976). Activities of citrate synthase and NAD+‐linked and NADP+‐linked isocitrate dehydrogenase in muscle from vertebrates and invertebrates. The Biochemical Journal, 154, 689–700. 10.1042/bj1540689 8036 PMC1172771

[phy270562-bib-0004] Apro, W. , Moberg, M. , Hamilton, D. L. , Ekblom, B. , Rooyackers, O. , Holmberg, H. C. , & Blomstrand, E. (2015). Leucine does not affect mechanistic target of rapamycin complex 1 assembly but is required for maximal ribosomal protein s6 kinase 1 activity in human skeletal muscle following resistance exercise. The FASEB Journal, 29, 4358–4373. 10.1096/fj.15-273474 26169935

[phy270562-bib-0005] Bennike, T. B. , Kastaniegaard, K. , Padurariu, S. , Gaihede, M. , Birkelund, S. , Andersen, V. , & Stensballe, A. (2016). Comparing the proteome of snap frozen, RNAlater preserved, and formalin‐fixed paraffin‐embedded human tissue samples. EuPA Open Proteomics, 10, 9–18. 10.1016/j.euprot.2015.10.001 29900094 PMC5988570

[phy270562-bib-0006] Blackwood, S. J. , Horwath, O. , Moberg, M. , Pontén, M. , Apró, W. , Ekblom, M. M. , Larsen, F. J. , & Katz, A. (2022). Extreme variations in muscle fiber composition enable detection of insulin resistance and excessive insulin secretion. The Journal of Clinical Endocrinology and Metabolism, 107, e2729–e2737. 10.1210/clinem/dgac221 35405014

[phy270562-bib-0007] Bottinelli, R. , Schiaffino, S. , & Reggiani, C. (1991). Force‐velocity relations and myosin heavy chain isoform compositions of skinned fibres from rat skeletal muscle. The Journal of Physiology, 437, 655–672. 10.1113/jphysiol.1991.sp018617 1890654 PMC1180069

[phy270562-bib-0008] Bull, H. B. , & Breese, K. (1978). Interaction of alcohols with proteins. Biopolymers, 17, 2121–2131.

[phy270562-bib-0009] Damer, A. , El Meniawy, S. , McPherson, R. , Wells, G. , Harper, M. E. , & Dent, R. (2022). Association of muscle fiber type with measures of obesity: A systematic review. Obesity Reviews, 23, e13444. 10.1111/obr.13444 35293095

[phy270562-bib-0010] Damsteegt, E. L. , McHugh, N. , & Lokman, P. M. (2016). Storage by lyophilization—Resulting RNA quality is tissue dependent. Analytical Biochemistry, 511, 92–96. 10.1016/j.ab.2016.08.005 27515991

[phy270562-bib-0011] Deshmukh, A. S. , Steenberg, D. E. , Hostrup, M. , Birk, J. B. , Larsen, J. K. , Santos, A. , Kjobsted, R. , Hingst, J. R. , Scheele, C. C. , Murgia, M. , Kiens, B. , Richter, E. A. , Mann, M. , & Wojtaszewski, J. F. P. (2021). Deep muscle‐proteomic analysis of freeze‐dried human muscle biopsies reveals fiber type‐specific adaptations to exercise training. Nature Communications, 12, 304. 10.1038/s41467-020-20556-8 PMC7803955 33436631

[phy270562-bib-0012] Edman, S. , Soderlund, K. , Moberg, M. , Apro, W. , & Blomstrand, E. (2019). mTORC1 signaling in individual human muscle fibers following resistance exercise in combination with intake of essential amino acids. Frontiers in Nutrition, 6, 96. 10.3389/fnut.2019.00096 31294029 PMC6603157

[phy270562-bib-0013] Edström, L. , & Kugelberg, E. (1968). Histochemical composition, distribution of fibres and fatiguability of single motor units. Anterior tibial muscle of the rat. Journal of Neurology, Neurosurgery, and Psychiatry, 31(5), 424–433. 10.1136/jnnp.31.5.424 5709826 PMC496396

[phy270562-bib-0014] Essen, B. , Jansson, E. , Henriksson, J. , Taylor, A. W. , & Saltin, B. (1975). Metabolic characteristics of fibre types in human skeletal muscle. Acta Physiologica Scandinavica, 95, 153–165. 10.1111/j.1748-1716.1975.tb10038.x 242187

[phy270562-bib-0015] Flockhart, M. , Nilsson, L. C. , Tais, S. , Ekblom, B. , Apró, W. , & Larsen, F. J. (2021). Excessive exercise training causes mitochondrial functional impairment and decreases glucose tolerance in healthy volunteers. Cell Metabolism, 33, 957–970. 10.1016/j.cmet.2021.02.017 33740420

[phy270562-bib-0016] Galpin, A. J. , Raue, U. , Jemiolo, B. , Trappe, T. A. , Harber, M. P. , Minchev, K. , & Trappe, S. (2012). Human skeletal muscle fiber type specific protein content. Analytical Biochemistry, 425, 175–182. 10.1016/j.ab.2012.03.018 22469996 PMC3358799

[phy270562-bib-0017] Goldstein, D. B. (1986). Effect of alcohol on cellular membranes. Annals of Emergency Medicine, 15, 1013–1018. 10.1016/s0196-0644(86)80120-2 3526990

[phy270562-bib-0018] Harris, R. C. , Hultman, E. , & Nordesjo, L. O. (1974). Glycogen, glycolytic intermediates and high‐energy phosphates determined in biopsy samples of musculus quadriceps femoris of man at rest. Methods and variance of values. Scandinavian Journal of Clinical and Laboratory Investigation, 33, 109–120. 4852173

[phy270562-bib-0019] Hernelahti, M. , Tikkanen, H. O. , Karjalainen, J. , & Kujala, U. M. (2005). Muscle fiber‐type distribution as a predictor of blood pressure: A 19‐year follow‐up study. Hypertension, 45, 1019–1023. 10.1161/01.Hyp.0000165023.09921.34 15837823

[phy270562-bib-0020] Horwath, O. , Edman, S. , Andersson, A. , Larsen, F. J. , & Apró, W. (2022). THRIFTY: A novel high‐throughput method for rapid fibre type identification of isolated skeletal muscle fibres. The Journal of Physiology, 600, 4421–4438. 10.1113/jp282959 36069036 PMC9825974

[phy270562-bib-0021] Horwath, O. , Moberg, M. , Hirschberg, A. L. , Ekblom, B. , & Apró, W. (2022). Molecular regulators of muscle mass and mitochondrial remodeling are not influenced by testosterone administration in young women. Frontiers in Endocrinology, 13, 874748. 10.3389/fendo.2022.874748 35498440 PMC9046720

[phy270562-bib-0022] Jonsson, W. O. , Ponette, J. , Horwath, O. , Rydenstam, T. , Soderlund, K. , Ekblom, B. , Azzolini, M. , Ruas, J. L. , & Blomstrand, E. (2022). Changes in plasma concentration of kynurenine following intake of branched‐chain amino acids are not caused by alterations in muscle kynurenine metabolism. American Journal of Physiology. Cell Physiology, 322, C49–C62. 10.1152/ajpcell.00285.2021 34817270

[phy270562-bib-0023] Kasahara, T. , Miyazaki, T. , Nitta, H. , Ono, A. , Miyagishima, T. , Nagao, T. , & Urushidani, T. (2006). Evaluation of methods for duration of preservation of RNA quality in rat liver used for transcriptome analysis. The Journal of Toxicological Sciences, 31, 509–519. 10.2131/jts.31.509 17202763

[phy270562-bib-0024] Kruse, C. P. S. , Basu, P. , Luesse, D. R. , & Wyatt, S. E. (2017). Transcriptome and proteome responses in RNAlater preserved tissue of Arabidopsis thaliana. PLoS One, 12, e0175943. 10.1371/journal.pone.0175943 28423006 PMC5397022

[phy270562-bib-0025] Lillioja, S. , Young, A. A. , Culter, C. L. , Ivy, J. L. , Abbott, W. G. , Zawadzki, J. K. , Yki‐Jarvinen, H. , Christin, L. , Secomb, T. W. , & Bogardus, C. (1987). Skeletal muscle capillary density and fiber type are possible determinants of in vivo insulin resistance in man. The Journal of Clinical Investigation, 80, 415–424. 10.1172/JCI113088 3301899 PMC442253

[phy270562-bib-0026] Lowry, O. , & Passonneau, J. (1972). A flexible system of enzymatic analysis. Academic Press.

[phy270562-bib-0027] Marin, P. , Andersson, B. , Krotkiewski, M. , & Bjorntorp, P. (1994). Muscle fiber composition and capillary density in women and men with NIDDM. Diabetes Care, 17, 382–386. 10.2337/diacare.17.5.382 8062604

[phy270562-bib-0028] Meijer, A. E. , Benson, D. , & Scholte, H. R. (1977). The influence of freezing and freeze‐drying of tissue specimens on enzyme activity. Histochemistry, 51, 297–303. 10.1007/BF00494365 870461

[phy270562-bib-0029] Molnar, A. , Lakat, T. , Hosszu, A. , Szebeni, B. , Balogh, A. , Orfi, L. , Szabo, A. J. , Fekete, A. , & Hodrea, J. (2021). Lyophilization and homogenization of biological samples improves reproducibility and reduces standard deviation in molecular biology techniques. Amino Acids, 53, 917–928. 10.1007/s00726-021-02994-w 34002278 PMC8128086

[phy270562-bib-0030] Mutter, G. L. , Zahrieh, D. , Liu, C. , Neuberg, D. , Finkelstein, D. , Baker, H. E. , & Warrington, J. A. (2004). Comparison of frozen and RNALater solid tissue storage methods for use in RNA expression microarrays. BMC Genomics, 5, 88. 10.1186/1471-2164-5-88 15537428 PMC534099

[phy270562-bib-0031] Raue, U. , Begue, G. , Minchev, K. , Jemiolo, B. , Gries, K. J. , Chambers, T. , Rubenstein, A. , Zaslavsky, E. , Sealfon, S. C. , Trappe, T. , & Trappe, S. (2024). Fast and slow muscle fiber transcriptome dynamics with lifelong endurance exercise. Journal of Applied Physiology (1985), 136, 244–261. 10.1152/japplphysiol.00442.2023 PMC11219013 38095016

[phy270562-bib-0032] Schiaffino, S. , & Reggiani, C. (2011). Fiber types in mammalian skeletal muscles. Physiological Reviews, 91, 1447–1531. 10.1152/physrev.00031.2010 22013216

[phy270562-bib-0033] Tanner, C. J. , Barakat, H. A. , Dohm, G. L. , Pories, W. J. , MacDonald, K. G. , Cunningham, P. R. , Swanson, M. S. , & Houmard, J. A. (2002). Muscle fiber type is associated with obesity and weight loss. American Journal of Physiology ‐ Endocrinology and Metabolism, 282, E1191–E1196. 10.1152/ajpendo.00416.2001 12006347

[phy270562-bib-0034] Tobias, I. S. , & Galpin, A. J. (2020). Moving human muscle physiology research forward: An evaluation of fiber type‐specific protein research methodologies. American Journal of Physiology. Cell Physiology, 319, C858–C876. 10.1152/ajpcell.00107.2020 32783659

[phy270562-bib-0035] Tobias, I. S. , Lazauskas, K. K. , Arevalo, J. A. , Bagley, J. R. , Brown, L. E. , & Galpin, A. J. (2018). Fiber type‐specific analysis of AMPK isoforms in human skeletal muscle: Advancement in methods via capillary nanoimmunoassay. Journal of Applied Physiology (1985), 124, 840–849. 10.1152/japplphysiol.00894.2017 29357518

[phy270562-bib-0036] Tobias, I. S. , Lazauskas, K. K. , Siu, J. , Costa, P. B. , Coburn, J. W. , & Galpin, A. J. (2020). Sex and fiber type independently influence AMPK, TBC1D1, and TBC1D4 at rest and during recovery from high‐intensity exercise in humans. Journal of Applied Physiology (1985), 128, 350–361. 10.1152/japplphysiol.00704.2019 31895596

[phy270562-bib-0037] van Eijsden, R. G. , Stassen, C. , Daenen, L. , Van Mulders, S. E. , Bapat, P. M. , Siewers, V. , Goossens, K. V. , Nielsen, J. , Delvaux, F. R. , Van Hummelen, P. , Devreese, B. , & Willaert, R. G. (2013). A universal fixation method based on quaternary ammonium salts (RNAlater) for omics‐technologies: Saccharomyces cerevisiae as a case study. Biotechnology Letters, 35, 891–900. 10.1007/s10529-013-1163-0 23417260

[phy270562-bib-0038] Vesali, R. F. , Klaude, M. , Rooyackers, O. E. , Tjäder, I. , Barle, H. , & Wernerman, J. (2002). Longitudinal pattern of glutamine/glutamate balance across the leg in long‐stay intensive care unit patients. Clinical Nutrition, 21, 505–514. 10.1054/clnu.2002.0583 12468371

[phy270562-bib-0039] Wu, Y. , Wu, M. , Zhang, Y. , Li, W. , Gao, Y. , Li, Z. , Wang, Z. , Lubec, G. , & Zhang, C. (2012). Lyophilization is suitable for storage and shipment of fresh tissue samples without altering RNA and protein levels stored at room temperature. Amino Acids, 43, 1383–1388. 10.1007/s00726-011-1212-8 22215254

[phy270562-bib-0040] Wyckelsma, V. L. , Lindkvist, W. , Venckunas, T. , Brazaitis, M. , Kamandulis, S. , Paasuke, M. , Ereline, J. , Westerblad, H. , & Andersson, D. C. (2020). Kynurenine aminotransferase isoforms display fiber‐type specific expression in young and old human skeletal muscle. Experimental Gerontology, 134, 110880. 10.1016/j.exger.2020.110880 32068089

[phy270562-bib-0041] Yoshikawa, H. , Hirano, A. , Arakawa, T. , & Shiraki, K. (2012). Mechanistic insights into protein precipitation by alcohol. International Journal of Biological Macromolecules, 50, 865–871. 10.1016/j.ijbiomac.2011.11.005 22115717

[phy270562-bib-0042] Yuan, F. , Xiong, G. , Cohen, N. A. , & Cohen, A. S. (2017). Optimized protocol of methanol treatment for immunofluorescent staining in fixed brain slices. Applied Immunohistochemistry & Molecular Morphology, 25, 221–224. 10.1097/PAI.0000000000000293 26509907 PMC4848166

[phy270562-bib-0043] Zhu, Y. , Mullen, A. M. , Rai, D. K. , Kelly, A. L. , Sheehan, D. , Cafferky, J. , & Hamill, R. M. (2019). Assessment of RNAlater(®) as a potential method to preserve bovine muscle proteins compared with dry ice in a proteomic study. Food, 8, 60. 10.3390/foods8020060 PMC6406653 30764583

